# Current Treatment Strategies for Elderly Patients with Metastatic Colon Cancer

**DOI:** 10.7759/cureus.4713

**Published:** 2019-05-22

**Authors:** Muhammad Idrees, Mohamed Tejani

**Affiliations:** 1 Internal Medicine, Bassett Medical Center - Columbia University, Cooperstown, USA; 2 Oncology, University of Rochester, Rochester, USA

**Keywords:** metastatic disease, colon cancer, chemotherapy

## Abstract

Colon cancer is one of the most common cancers in the United States and is expected to rise as the prevalence of colon cancer is increasing with increasing aging population. Though some studies have shown benefits of chemotherapy in the elderly population, however, they are also at risk of drug toxicities. We searched major search engines including PubMed, Medline and EMBASE and reviewed articles published in the last 10 years. Here we present current treatment strategies available for the metastatic colon cancer in elderly patients.

## Introduction and background

Colon cancer is one of the most common cancers in the United States, as well as in the entire world, with almost 1.36 million new cases every year worldwide. It is estimated that there will be 101,420 new cases of colon cancer in 2019 in the US alone [1].

Most cancers occur in the elderly population. The median age of diagnosis for colon cancer is 69 years, with almost 70% of cases occurring in individuals more than 65 years of age and 40% of cases occurring in ages >75 years [1]. With an aging population, the prevalence of colon cancer in the population is going to increase. The elderly population who are frailer and at more risk of getting colon cancer have been under-researched in the past regarding appropriate treatment options for metastatic colon cancer. Moreover, colon cancer in the elderly has also been noted to have more BRAF V600E (B-RAF Proto-oncogene) mutations than early-onset colon cancer in young people, which is usually part of cancer syndrome. Older people have also been noted to harbor more MSI/CIMP mutations (microsatellite instability/CpG island methylator phenotype).

Chemotherapy is used in the treatment of colon cancer in the elderly population in adjuvant settings for Stage 3 cancers, including in palliative settings in metastatic cancer. Studies have shown improvement in progression-free survival and overall survival in both settings. However, elderly patients are also at the risk of experiencing more drug toxicity and requiring hospitalization. In this article, we will review current treatment strategies available for the metastatic colon cancer in elderly patients.

## Review

Methodology

We searched database including PubMed, Medline, and EMBASE using MeSH terms "Metastatic Colon Cancer", "Treatment and Elderly" and searched for articles which were clinical trials or review articles published in last 10 years, through January 2019. The initial searches resulted in 463 articles. Out of these articles, we selected ones which mentioned chemotherapy and immunotherapy in the treatment of metastatic colon cancer.

Active agents for metastatic colon cancer

Chemotherapy in metastatic cancer has been shown to improve outcomes over supportive care only. An analysis of patients over the age of 65 who received chemotherapy for metastatic colon cancer demonstrated a 6.8-month improvement in life expectancy between 1995 and 2005 that is possibly attributable to the introduction of newer chemotherapeutic agents [2]. Despite the demonstrated benefits of these new agents, the available evidence suggests that fewer elderly patients receive certain agents as a component of initial therapy as compared with younger individuals, presumably because of toxicity concerns. Here is a list of active agents for the treatment of metastatic colon cancer.

Single Agents

(a) 5-FU (fluorouracil)/Leucovorin: It is usually used in combination with irinotecan or oxaliplatin as standard therapy for metastatic colorectal cancer, however, it can be used alone in patients who can not tolerate triple therapy. This formulation is given in two forms; bolus versus continuous infusion. Studies have shown that bolus 5-FU is slightly more toxic than infusion form and causes more diarrhea and myelosuppression than continuous infusion 5-FU which is more likely to cause hand-foot syndrome and mucositis, especially in older patients (>70 years) [3-4]. Pooled analysis of 22 European clinical trials involving total 3825 patients, out of which 629 were over 70 years of age, showed that ‘fit’ elderly patients benefited at least to the same extent from palliative chemotherapy with 5-FU as younger patients. Also, infusional 5-FU was shown to be more effective than bolus 5-FU in both age groups [5].

Another retrospective cohort study involving 4768 patients over 65 years of age found similar findings that 5-fluorouracil adjuvant therapy is significantly associated with reduced mortality in older patients, similar to the association found in randomized controlled trials among younger patients [6]. Analysis of two clinical trials done by Chiara et al. showed that there was no significant difference in toxicity evident between patients older than or younger than 65 years [7].

(b) Capecitabine (Cap): Capecitabine (fluoropyrimidine carbamate), an orally administered chemotherapeutic agent, is a pro-drug that is converted to 5-FU following absorption. It is considered as an oral alternative to 5-FU as first-line therapy for the metastatic colorectal cancer. A population-based study conducted using the British Columbia registry involving two cohorts, one using 5-FU and other using capecitabine, showed that overall survival between the two-time cohorts did not differ significantly. The toxicities resulting in dose delay and/or reduction were comparable [8].

In metastatic colorectal cancer (MRC) FOCUS2 trial (FU, capecitabine, FU plus oxaliplatin as well as oxaliplatin plus capecitabine), capecitabine was noted to be equally effective as compared 5FU, albeit as more a toxic alternative. Common adverse effects were noted to be diarrhea and hand-foot syndrome [9-10]. However, in one other trial, capecitabine demonstrated an equal efficacy and safety profile superior to that of 5-FU/leucovorin, with a significantly lower incidence of adverse effects such as diarrhea [11].

(c) Irinotecan: Irinotecan, a topoisomerase I inhibitor, is used alone or in combination with 5-FU, as well as with targeted agents. It is used alone as well as in combination with other chemotherapy agents as a second-line agent. One clinical trial by Chau et al. showed similar efficacy of irinotecan monotherapy in 5-FU resistant metastatic colon cancer without excessive toxicity [12]. Common toxic effects were noted to be diarrhea and myelosuppression similar to previous chemotherapeutic agents [13].

(d) Oxaliplatin (Ox): Oxaliplatin is a platinum analog which is used in combination with 5-FU or capecitabine as a first-line regimen for metastatic colorectal cancer. It is not used as monotherapy. A significant adverse effect is peripheral neuropathy [14].

Combination Regimen

(a) FOLFOX (5-FU, leucovorin, oxaliplatin): FOLFOX has been shown to be effective and well tolerated in younger patients, however, MRC FOCUS2 trial showed that elderly patients can also tolerate the standard dose of FOLFOX [9]. Pooled analysis showed that FOLFOX was associated with a significantly higher objective response rate (38% versus 11%) and disease control rate (objective response plus stable disease, 71% versus 46%). There was a trend towards a longer median progression-free period (5.8 versus 3.5 months) and overall survival (10.7 versus 10.1 months) with FOLFOX, which was not statistically significant [15].

(b) FOLFILIRI (5FU, Leucovorin, irinotecan): Many clinical trials have shown that FOLFILIRI is well tolerated in elderly patients for example in a clinical trial by Aparicio et al. which studied toxicity in 166 patients more than 75 years old. This showed that the overall response rate was higher with FOLFIRI (42% versus 21%). However, irinotecan was also noted to have higher side effects, including febrile neutropenia and diarrhea [16]. In a subset analysis of older (≥ age 70) versus younger patients in randomized phase III BICC-C (bolus, infusional, or capecitabine with Camptosar-celecoxib) study, median progression-free and overall survival were both longer in the FOLFIRI group [17].

(c) CAPOX/XELOX (capecitabine/oxaliplatin): It is considered as a reasonable alternative to 5-FU-based chemotherapy regimens for metastatic colorectal cancer as studies have shown that it is equally efficacious, however, it is more toxic as compared to FOLFOX. A Spanish cooperative group for treatment of digestive tumors 03-TTD-01 (XELOX vs oxaliplatin + 5-FU CI) Phase III study included 109 patients, 70 years of age or older, and 233 younger individuals. The objective response rates for XELOX in older and younger patients were 35% and 45%, respectively, and median overall survival was 17 versus 21 months. XELOX group was noted to have higher toxicity [18]. Similarly, in MRC FOCUS2 trial, compared with FOLFOX, the use of XELOX was associated with a slightly lower objective response rate (32% versus 38%) and overall disease control rate (objective response plus stable disease, 65% versus 71%), identical progression-free survival (5.8 months in each group), and a trend toward longer median overall survival that was not statistically significant (12.4 versus 10.7 months) [9]. The overall risk of having a Grade 3 or worse adverse event was higher with XELOX than FOLFOX (43% versus 33%).

(d) XALIRI (capecitabine/irinotecan): Randomized BICC-C (bolus, infusional, or capecitabine with Camptosar-celecoxib) trial sub-analysis of elderly (less than 70 years of age) versus non-elderly patients enrolled in the trial, elderly patients had significantly higher rates of asthenia and dehydration with XELIRI compared with their younger counterparts [19]. Similar high rates of toxicity with XELIRI have been seen by others despite the use of lower starting doses of both drugs [20].

Anti-VEGF Factor

(a) Bevacizumab: It is a humanized monoclonal antibody targeting vascular endothelial growth factor (VEGF). Adding bevacizumab to first-line chemotherapy regimens containing a fluoropyrimidine, irinotecan or oxaliplatin has been shown to improve response rates, progression-free survival and overall survival as was noted in multi-center AVEX (bevacizumab plus capecitabine versus capecitabine alone in elderly patients with metastatic colorectal cancer) trial [21]. Common adverse effects include uncontrolled hypertension, stroke and delayed wound healing [22]. 

(b) Aflibercept: It is a recombinant fusion protein consisting of VEGF binding portions from key domains of human VEGF receptors 1 and 2 fused to the fragment crystallizable region (Fc region) of human immunoglobulin G1. The VELOUR trial (addition of aflibercept to FOLFIRI and placebo) showed that median overall survival was significantly longer in patients treated with aflibercept vs placebo + FOLFIRI alone (13.5 versus 12.1 months) [23]. Side effects were noted to be similar to bevacizumab.

(c) Ramucirumab (RAM): It is a monoclonal antibody that binds to the VEGFR-2 extracellular domain and prevents binding of all VEGF ligands. Ramucirumab has been shown to improve overall survival when used as second-line therapy with FOLIRI for metastatic colon cancer in RAISE (Ramucirumab versus placebo in combination with second-line FOLFIRI in patients with metastatic colorectal cancer that progressed during or after first-line therapy with bevacizumab, oxaliplatin, and a fluoropyrimidine) Phase III clinical trial [24].

Anti-EGFR Monoclonal Antibodies (Cetuximab and Panitumumab)

These are two monoclonal antibodies targeting the epidermal growth factor receptor (EGFR) which are used in RAS positive metastatic colon cancer. These are not effective in tumors which have BRAF V600E mutation. In one clinical trial involving a comparison of cetuximab in the elderly, it was noted that efficacy was similar to that expected in younger individuals and that tolerability of cetuximab was acceptable in elderly patients [25-27].

Receptor Tyrosine Kinase Inhibitor (Regorafenib)

Regorafenib is a new oral multikinase inhibitor that blocks the activity of several protein kinases, including the VEGF and EGFR pathways. It is approved as a single agent for the treatment of patients with refractory mCRC. CORRECT (colorectal cancer treated with regorafenib or placebo after failure of standard therapy) trial, which compared best supportive care plus regorafenib (160 mg orally once daily for three out of every four weeks) or placebo in 760 patients with chemotherapy-refractory disease, demonstrated a significant survival benefit for regorafenib (median 6.4 versus five months) [28]. The subgroup analysis of the CORRECT trial published in the American Society of Clinical Oncology (ASCO) in 2012 showed similar efficacy in elderly patients [29]. Common side effects reported in the CORRECT trial were fatigue, diarrhea, hand-foot skin reaction, and liver failure.

Trifluridine-tipiracil

Trifluridine-tipiracil was associated with a significant prolongation in median overall survival in progressive metastatic colon cancer in RECOURSE (Refractory Colorectal Cancer Study) phase III trial [30]. However, there are no published data on the safety or efficacy of trifluridine-tipiracil in the elderly.

*Immunotherapy* (*Nivolumab, Pembrolizumab, Ipilimumab*)

Immunotherapy drugs work as immune checkpoint inhibitors that target the programmed death receptor-1 (PD-1). They have been shown to be effective in metastatic colon cancer harboring microsatellite instability/deficient mismatch repair (MSI/dMMR) genes.

## Conclusions

Treating elderly patients with metastatic colon cancer has always been challenging due to fear of tolerability and side effects from chemotherapy. Studies have shown that elderly patients with good performance status benefit from chemotherapy equally as younger patients do, with comparable side effects. Regarding what chemotherapy agent would be the best for elderly patients depesnds on what are the expectations of the patient regarding chemotherapy and side effects. For patients with metastatic colon cancer who are willing to have intravenous chemotherapy, FOLFOX has been shown to be the best first-line therapy. When patients progress, FOLFOX can be changed to FOLFIRI, or vice-versa, while maintaining treatment with bevacizumab. If the patient has a K-ras wild-type tumor, cetuximab can be added to FOLFIRI, especially if a FOLFIRI-based regimen was not used first-line. If the patient has MSI/dMMR tumor, the patient can be given immunotherapy (nivolumab, pembrolizumab).
